# Live imaging of developmental processes in a living meristem of *Davidia involucrata* (Nyssaceae)

**DOI:** 10.3389/fpls.2014.00613

**Published:** 2014-11-13

**Authors:** Markus Jerominek, Kester Bull-Hereñu, Melanie Arndt, Regine Claßen-Bockhoff

**Affiliations:** ^1^Institut für Spezielle Botanik, Johannes Gutenberg-UniversitätMainz, Germany; ^2^Escuela de Pedagogía en Biología y Ciencias, Universidad Central de ChileSantiago de Chile, Chile; ^3^Facultad de Ciencias Biológicas, Pontificia Universidad Católica de ChileSantiago de Chile, Chile

**Keywords:** live imaging, *in vivo*, morphogenesis, floral unit meristem (FU meristem), epi-illumination light microscopy (ELM), *Davidia involucrata*, Nyssaceae

## Abstract

Morphogenesis in plants is usually reconstructed by scanning electron microscopy and histology of meristematic structures. These techniques are destructive and require many samples to obtain a consecutive series of states. Unfortunately, using this methodology the absolute timing of growth and complete relative initiation of organs remain obscure. To overcome this limitation, an *in vivo* observational method based on Epi-Illumination Light Microscopy (ELM) was developed and tested with a male inflorescence meristem (floral unit) of the handkerchief tree *Davidia involucrata* Baill. (Nyssaceae). We asked whether the most basal flowers of this floral unit arise in a basipetal sequence or, alternatively, are delayed in their development. The growing meristem was observed for 30 days, the longest live observation of a meristem achieved to date. The sequence of primordium initiation indicates a later initiation of the most basal flowers and not earlier or simultaneously as SEM images could suggest. *D. involucrata* exemplarily shows that live-ELM gives new insights into developmental processes of plants. In addition to morphogenetic questions such as the transition from vegetative to reproductive meristems or the absolute timing of ontogenetic processes, this method may also help to quantify cellular growth processes in the context of molecular physiology and developmental genetics studies.

## Introduction

One of the most significant technical advances of the last decades in plant sciences is the *in vivo* observation of developmental processes. *In vivo* techniques have the great advantage that they are non-destructive and allow imaging of phenomena as they occur within the plant body (Grandjean et al., 2004; Sijacic and Liu, 2010; Hiroi et al., 2013). Several innovations in light microscopy and fluorescence labeling technologies have offered amazing insights into developmental processes in meristematic tissues (Campilho et al., 2006; Reddy, 2008).

**Figure 1:**
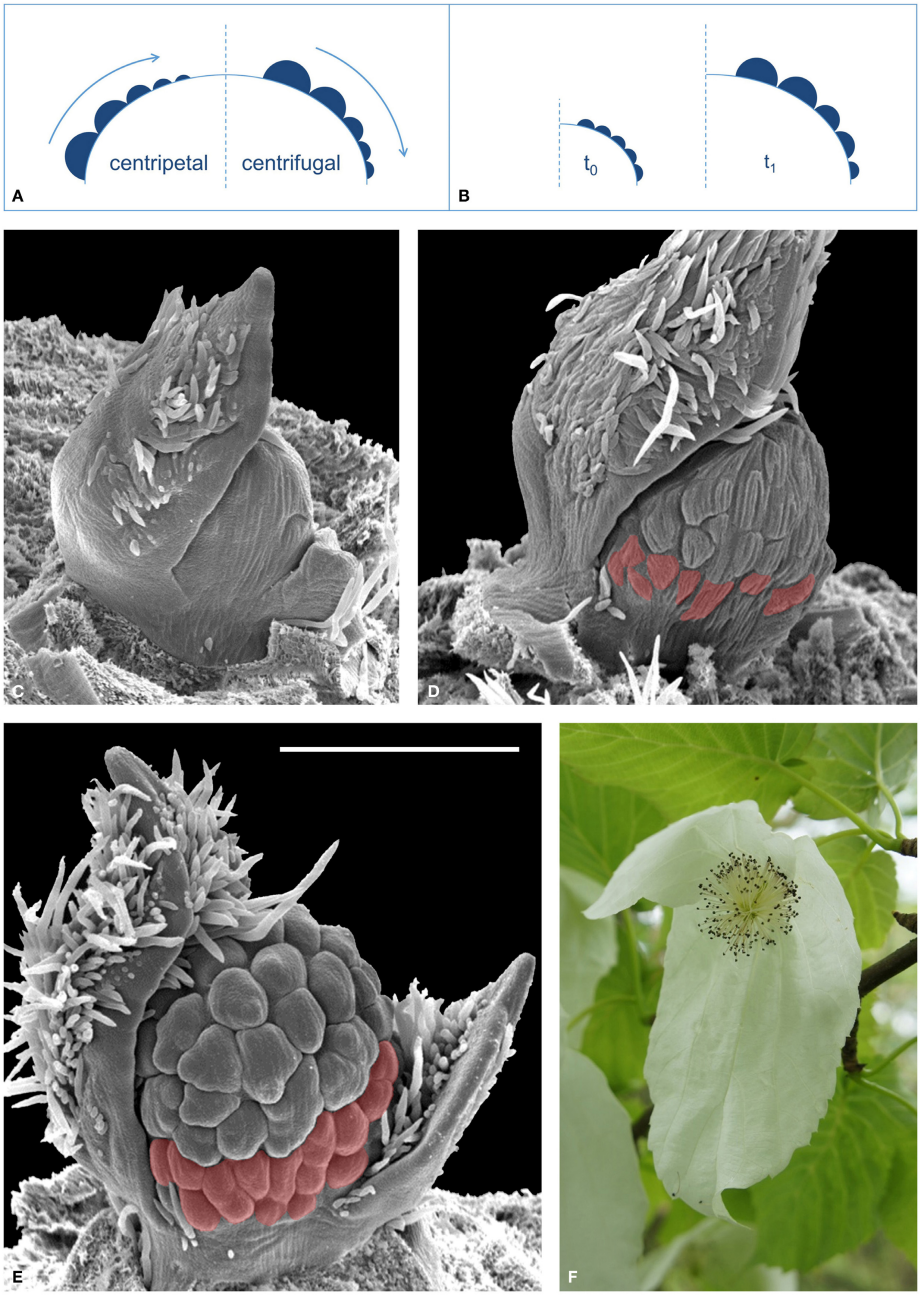
**Development of reproductive meristems. (A)** The relative size of flower primordia (filled semicircles) of a reproductive meristem (large dome) is generally used to reconstruct the sequence of initiation (arrow), which either occurs toward the center, i.e., “centripetal” (left sketch), or toward the flanks, i.e., “centrifugal” (right sketch). **(B)** Different growing rates of simultaneously initiated flower primordia lead to a false interpretation of the initiation sequence (here centrifugal). **(C–E)**, SEM images of a developing FU of *Davidia involucrata*: **(C)** Young meristem starting to fractionate flower primordia. **(D)** Later stage, with many flower primordia initiated. Note that the most basal primordia (red) are smaller. **(E)** A male FU meristem with stamen initiation in equatorial primorida and delayed development of the most basal flowers (red). All SEM images are in the same scale, bar line = 500 μm. **(F)** Male inflorescence (FU) of *D. involucrata* subtended by two conspicuous extrafloral bracts.

While these approaches primarily address gene expression or hormone flux issues (Grandjean et al., 2004; Heisler et al., 2005; Vernoux et al., 2011), traditional imaging techniques such as histology, scanning electron microscopy (SEM), epi-illumination light microscopy (ELM) and computer tomography (CT, Staedler et al., 2013) have succeeded in providing clear information regarding morphogenesis at the tissue level. Unfortunately, these techniques are normally destructive and necessarily imply the observation of many individuals in different developmental states to reconstruct ontogenetic sequences. Thus, this approach demands some interpretation, since *the same* developing structure is not being observed among different samples. Particularly, this can become a complex issue when reconstructing the development of numerically variable structures, e.g., condensed inflorescences known as “floral units” (Claßen-Bockhoff and Bull-Hereñu, 2013). In floral units (FU), flower primordia usually fractionate in either a centripetal [e.g., umbellets in Apiaceae, (Bull-Hereñu and Claßen-Bockhoff, 2010)] or centrifugal sequence [e.g., cyathia in *Euphorbia*, (Prenner and Rudall, 2007)] (Figure 1A). If primordia appear almost simultaneously the sequence is interpreted from the arrangement and size of flower primordia, assuming that the smaller ones have been initiated later (Figure 1A). A direct size-age correlation is used because of the *a priori* assumption that all flower primordia share similar growth rates. However, simultaneous initiation of primordia but a slower growth rate in the basal primordia would create a false impression of centrifugal initiation (Figure 1B).

This is a case where traditional imaging techniques find their interpretational limit when trying to elucidate which ontogenetic process is actually occurring.

The floral unit of *Davidia involucrata* Bail. (Nyssaceae, Figure 1F) illustrates this conflict. During development, a number of flower primordia arise almost simultaneously on the FU meristem (Figure 1C); those in the equatorial zone are larger than the most basal ones (Figures 1D,E). As stated above it remains unclear whether the basal flower primordia in *D. involucrata* originate later, i.e., in a centrifugal sequence, or grow more slowly (Figure 1B). This problem can only be solved by *in vivo* observation of the same growing meristem. By developing an automated ELM live imaging technique it was possible to record the development of a male floral unit meristem of *D. involucrata* for a period of 30 days.

## Materials and methods

### Plant material

**Figure 2:**
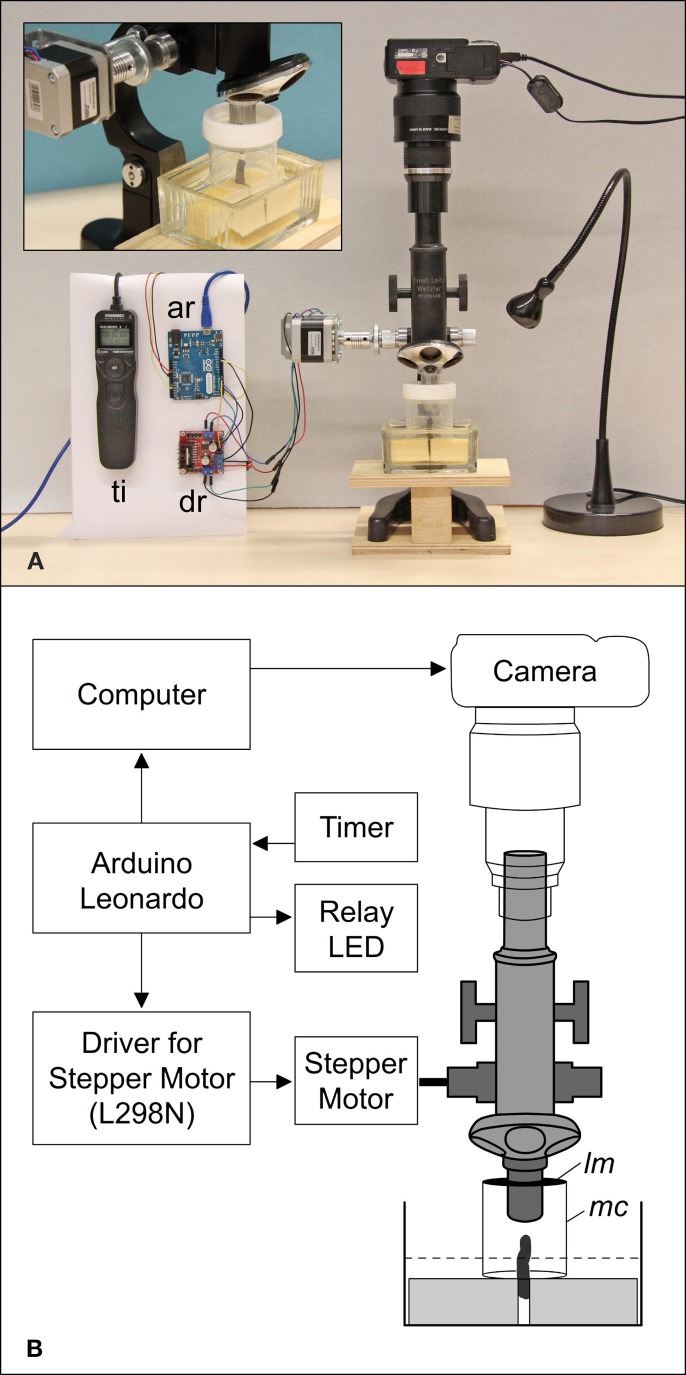
**Setup (A) and schematic diagram (B) of the automated arrangement**. A branch of *Davidia involucrata* is placed in a staining dish with tap water and fixed with two styrofoam blocks **(A**, Close-up). The tip of the branch (meristem) is protected by a modified probe tube forming a moisture chamber (mc). The upper side is covered by a latex membrane (lm) that envelops the objective lens. The lower side is closed by the water level (dotted line). ar, Arduino Leonardo (microcontroller); dr, driver for stepper motor; lm, latex membrane; mc, moisture chamber; ti, timer remote control.

A FU meristem of *D. involucrata* was taken from a tree cultivated in the Botanical Garden at the University of Mainz, Germany. The meristem, located at the tip of a short branch, was dissected and mounted with styrofoam in a histological staining dish while the site of fracture was submerged in tap water (Figure 2). To prevent dehydration the meristem was directly covered by a modified probe tube (50 ml) that was used as moisture chamber. The lower side was closed by the water of the staining dish and the upper side was covered with a latex membrane that enveloped the objective lens. During observation, the water in the staining dish was checked and refilled twice a week.

### Epi-illumination light microscopy (ELM)

**Figure 3:**
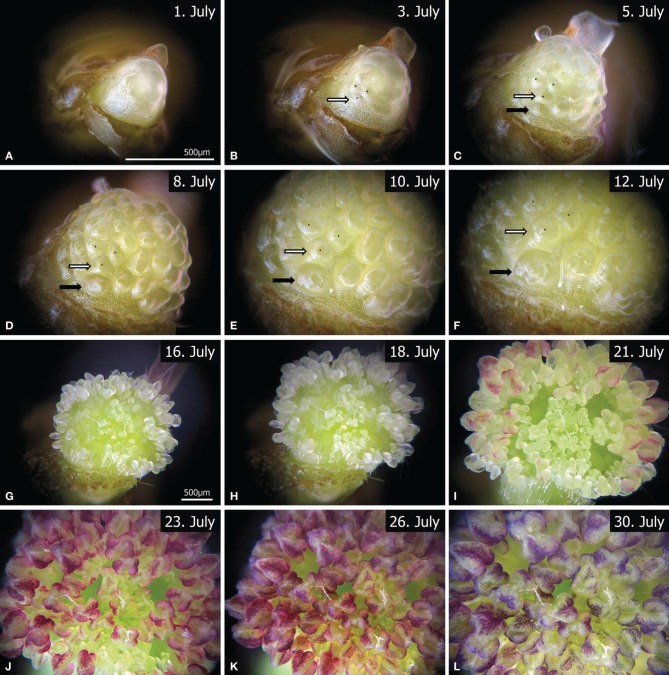
**Twelve representative images from the time-lapse videos (Video S1, S2), showing the development of a male FU, from flower (A–D) and stamen initiation (EF) to anther development (G–K)**. Due to meristem growth the magnification was changed between **(A–F)** (Video S1) and **(G–L)** (Video S2). The corresponding bar lines are indicated in **(A,G)**. The white and black arrows mark two individual primordia at different developmental states. The outer primordia (black arrow) are clearly initiated later than the inner ones (white arrow). Videos available at www.spinningspecies.com/davidia.html.

Since common binocular microscopes have angular optical paths that produce a shift of images in a stack, we used a monocular microscope (Leitz Wetzlar, Figure 2). Photographs were taken with a Canon Powershot G9 that was mounted on the eyepiece and triggered by a computer (Canon CameraWindow). For an automated stacking procedure a stepper motor was adapted to the focus wheel of the microscope and controlled by a four-channel motor driver circuit (L298N). For illumination a LED light was switched on during the image capture process. The whole stacking cycle was controlled by an open-source microcontroller (Arduino Leonardo) that was connected to the motor driver to change the focus. The camera was triggered by an implemented keyboard source code via a computer and the LED was controlled via a relay. Each cycle began with the activation of the LED and was followed by raising the microscope to the highest focus point. The next two steps that triggered the camera and moved the microscope to a lower focus position were repeated until the lowest focus point was reached (70 times). To finish the cycle the LED was turned off. For live imaging the cycle was triggered and repeated once per hour by a timer (remote control for cameras). Since the meristem grew, the focus range was checked twice a week and manually readjusted. After the meristem became too large the magnification was reduced from a 10-fold to a 4-fold objective (comp. Figures 3A–F and G–L). Accordingly, live observation is divided into two time lapse videos.

### Image processing

To generate a time-lapse video all images from each stack had to be combined to one single sharp image (Wilson et al., 2006). This image processing was performed with CombineZP (http://www.hadleyweb.pwp.blueyonder.co.uk/), which allows automation of this process for all image stacks (CZBatch.exe). Here the soft stack algorithm was applied. To compensate the shift of photographs due to image processing as well as to the readjustments of the focus range, all images were arranged by adjusting and shifting them consecutively with Amira (Mercury Computer Systems). The resulting time-lapse videos were generated either with JPGVideo (http://www.ndrw.co.uk/) or by loading all images into a flash movie (Adobe Flash CS3, www.spinningspecies.com/davidia.html).

## Results and discussion

### Meristem development

The observation began with an undifferentiated meristem (Figure 3A, Video S1). Flower initiation was clearly seen on the 3rd day of observation (Figure 3B), when almost all flower primordia appeared more or less simultaneously. After 2 days and a considerable enlargement of the FU meristem, additional primordia arose at its base (Figure 3C, black arrow). In contrast to the SEM sequence (Figures 1C,D) the most basal primordia develop faster, since they became larger than the ones in the equatorial zone as the FU meristem continued to expand during the next 5 days (Figures 3D,E). At day 12 the first anther primordia could be observed in the basal flowers (Figure 3F). These anthers developed faster than those of the upper flowers (Figures 3G–I, Video S2). Finally, the anthers covered the whole meristem and became red (Figures 3J,K) to purple (Figure 3L).

The observation of the male FU of *D. involucrata* performed here indicates that the smaller size of the proximal flower primordia may be due to basipetal initiation. The initiation of ray florets in Asteraceae heads exhibits a similar phenomenon; SEM studies suggested that they emerged later (Harris et al., 1991; Harris, 1995; Bello et al., 2013). However, the alternative “basal delay” hypothesis discussed here cannot be rejected until an *in vivo* observation of a head meristem is performed.

### Live imaging with ELM

Our study shows that ELM can be used to obtain detailed structural information of a growing meristem. This technique could be an alternative to replica molds (Williams and Green, 1988; Green et al., 1991; Dumais and Kwiatkowska, 2001; Kwiatkowska, 2006; Barabé et al., 2007; Szczęsny et al., 2009) and confocal microscopy (Grandjean et al., 2004; Heisler et al., 2005; Campilho et al., 2006; Reddy, 2008; Sijacic and Liu, 2010; Vernoux et al., 2011), the only imaging techniques that have been successful in obtaining data from the development of a single meristem up to now. In contrast to conventional methods, *in vivo* techniques are able to analyze developmental timing and clear up relative initiation issues (Table 1). While SEM only allows reconstruction of developmental steps, live imaging reveals the absolute time of organ initiation and duration of growth. Moreover, this low-cost method is compatible with color imaging and is simple: objects do not need to be fixed, critical point dried or sputtered.

**Table 1:** **Comparison of different visualizing techniques used in plant development**.

**Technique**	**Pros and Cons**
Histology	+ Reveals inner structures
	+ Reveals gene expression patterns
	− Destructive
SEM	+ Retrieves high structural details
	− Destructive
Confocal microscopy	+ *In vivo* observation
	+ Reveals gene expression patterns
	− High cost
	− Demands appropriate culturing methods
	− Fluorescent labeling required
Replica molds	+ *In vivo* observation
	+ Retrieves high structural details
	− Time-consuming and limited application
Micro-CT	+ Reveals inner structures
	− High cost and time-consuming
	− Destructive
ELM	+ Inexpensive
	+ Color imaging
	− Destructive
Live-ELM	+ Inexpensive
	+ Color imaging
	+ *In vivo* observation
	− Complex image processing

Although ELM has been successfully used for fixed material (Bartlett et al., 2008; Dadpour et al., 2008, 2011, 2012; Oraei et al., 2013), live ELM imaging of a developing meristem has been largely neglected in the past. The main reason may have been the difficulty in maintaining the tissue healthy for several days.

The spatial resolution of ELM depends on the properties of the microscope and the stepper motor. Thereby the depth of field depends on the aperture of the objective for a single image and on the range of the fine focus for the whole stack. The distance between single images depends on the physical resolution of the stepper motor. We used 200 steps per revolution which provided a resolution of 1 μm, but this could be made smaller by driving the motor with micro-steps.

The temporal resolution of the system is defined by the duration of a stack, which depends on the speed of the motor and the exposure time of the camera. Since we used a long exposure time (1 s) and a low motor speed, the stacking procedure took about 15 min. Another serious problem was the correct illumination of the object. To avoid reflections that would hamper the stacking procedure we used a weak indirect illumination of the object with long exposure times. Therefore, the light cone was directed to the upper latex membrane (Figure 3B, lm).

## Conclusions

By means of this automated live ELM system it is possible to create real-time high resolution records of growing plant meristems. The methodology illustrated here can contribute substantially to ontogenetic plant imaging, as it provides a potential easy-to-operate tool for monitoring and quantification of growth processes. On the morphogenetic level it can give new insights into organ growth, meristematic transitions, absolute timing of plant development and morphometric responses to experimental stimuli (Reinhardt et al., 2003; Bello et al., 2013; Claßen-Bockhoff and Bull-Hereñu, 2013). On the cellular level it may help to analyze cell expansion and division in the context of molecular physiology and developmental genetic studies (Hamant et al., 2010; Sijacic and Liu, 2010; Uyttewaal et al., 2012; Sassi and Vernoux, 2013).

## Author contributions

Markus Jerominek and Kester Bull-Hereñu initiated the study. Markus Jerominek designed the experimental setup and drafted the manuscript. Melanie Arndt provided the SEM data of FU development in *Davidia involucrata*, supervised by Regine Claßen-Bockhoff. Kester Bull-Hereñu and Regine Claßen-Bockhoff analyzed the data and contributed to the final manuscript.

### Conflict of interest statement

The authors declare that the research was conducted in the absence of any commercial or financial relationships that could be construed as a potential conflict of interest.

## Abbreviations

SEM: scanning electron microscopy
ELM: epi-illumination light microscopy
FU: floral unit.
